# Employees’ Longer Working Lives in Europe: Drivers and Barriers in Companies

**DOI:** 10.3390/ijerph17051658

**Published:** 2020-03-04

**Authors:** Andrea Principi, Jürgen Bauknecht, Mirko Di Rosa, Marco Socci

**Affiliations:** 1 Centre for Socio-Economic Research on Ageing, IRCCS INRCA – National Institute of Health and Science on Ageing, 60124 Ancona, Italy; a.principi@inrca.it (A.P.); m.socci@inrca.it (M.S.); 2 Fliedner Fachhochschule Düsseldorf – University of Applied Sciences, 40489 Düsseldorf, Germany; bauknecht@fliedner-fachhochschule.de; 3 Scientific Direction, IRCCS INRCA – National Institute of Health and Science on Ageing, 60124 Ancona, Italy

**Keywords:** older workers, age management, extending working life, employers, international study

## Abstract

This paper identifies, within companies’ sectors of activity, predictors of Human Resource (HR) policies to extend working life (EWL) in light of increasing policy efforts at the European level to extend working life. Three types of EWL practices are investigated: the prevention of early retirement (i.e., encouraging employees to continue working until the legal retirement age); delay of retirement (i.e., encouraging employees to continue working beyond the legal retirement age); and, recruitment of employees who are already retired (i.e., unretirement). A sample of 4624 European organizations that was stratified by size and sector is analyzed in six countries. The main drivers for companies’ EWL practices are the implementation of measures for older workers to improve their performance, their working conditions, and to reduce costs. In industry, the qualities and skills of older workers could be more valued than in other sectors, while the adoption of EWL practices might be less affected by external economic and labor market factors in the public sector. Dutch and Italian employers may be less prone than others to extend working lives. These results underline the importance of raising employers’ awareness and increase their actions to extend employees’ working lives by adopting age management initiatives, especially in SMEs, and in the services and public sectors.

## 1. Introduction

Population aging and the need to guarantee pay-as-you-go pension system sustainability and a sufficient supply of skilled labor are the main reasons for European policies that are aimed at extending working life (EWL) [1]. At the macro level, reforms in various policy fields mainly manage this issue [2]. At the individual level, the decision to retire or not is supposed to be managed based on voluntary choice [3]. However, the company level is the key level where the extension of working life materializes or otherwise [4]. Indeed, companies might not want to implement measures to pursue this policy aim [5], may implement measures that strengthen or weaken this goal promoted at the macro level, or have no effect at all [6]. Furthermore, companies could influence the micro level by giving employees little leeway regarding their retirement [4].

Studies suggest that employers tend not to invest in older workers [5]. However, the organizational level remains largely unexplored [7], especially concerning the specific issue of the actual provision of opportunities for prolonging employees’ working lives. Some previous studies investigated HR practices towards older workers in terms of employability [8] or general recruitment behavior or retention strategies [9]. However, these studies did not allow for the possibility to demonstrate the concrete effectiveness of investigated strategies on the lengthened working life of employees, and the latter was never investigated within the sectors of activity, with few exceptions [10]. The present study specifically concentrates on HR policies, which, if implemented, are connected to the concrete lengthening of the working lives of older employees. It contributes to the state of research on the main drivers of and barriers to the extension of working life via labor demand, by exploring, within the sectors of activity: the prevention of early retirement (i.e., encouraging employees to continue working until the legal retirement age); postponement of retirement (i.e., encouraging employees to continue working beyond the legal retirement age); and, unretirement (i.e., the recruitment of employees who are already retired), that is, when an individual fully retires and then returns to employment [11].

### 1.1. Conceptual Framework and Hypotheses

This study’s theoretical framework is based on the overall hypothesis that the behavior of employers with regard to prolonging older employees’ working lives, rests on their assessment of labor costs against labor productivity [12]. It has been observed that workforce aging might imply negative consequences in terms of productivity, e.g., due to age-related decline in physical and some cognitive abilities [13]. Furthermore, the costs may increase due to the high seniority-based wages of older workers [14]. However, the participation of older employees in the labor market is increasing [15], and employers need to find new strategies for dealing with workforce aging, which aimed at increasing productivity and reducing costs.

The relationship between older workers’ (decline in) productivity and (increase in) costs could also depend on the company´s sector. Employers could be less inclined to prolong the working lives of their employees than in others in certain sectors with age-specific wage-productivity gaps. Therefore, we differentiate between sectors (industry, private services, and public sector). We identified four main areas within which employers deal with the relationship between costs and productivity against the background of staff aging based on the evidence available in the literature: the implementation of HR policies, workforce characteristics, company structural characteristics, and the position of the company in the economic and labor market climate.

#### 1.1.1. Human Resource Policies

The labor productivity of older workers can be boosted, according to human capital theory [16], thus it can be better aligned to higher remuneration in the final stage of the working career [14], through investment in human capital. This would make it possible to avoid the latter’s deterioration due to possible skill obsolesce and/or worsened physical and mental health. Examples of HR policies aiming to increase the human capital of older workers include: decrease of the workload, training programs, career planning, ergonomic measures and workplace design, part-time work, and demotion [5]. Still, some measures could have negative impacts for older workers, e.g., demotion could reduce work motivation [17]. Furthermore, partial retirement by reducing the number of working hours until retirement might indicate a wish to dismiss older workers, rather than retain them [18]. Although early retirement schemes cannot be considered measures aimed at increasing human capital, we are interested in exploring their effect, since these schemes could foster work after retirement, in the employers’ attempt to balance costs and productivity by reducing the costs [19].

**H1a:**  *In general, companies implementing HR policies to manage the relationship between costs and productivity of older workers are more likely to extend the latter’s working lives.*

**H1b:**  *As regards exit policies, companies implementing this measure are more likely to extend the working life through unretirement.*

#### 1.1.2. Workforce Characteristics

The literature documents negative stereotypes that influence employers with regard to the productivity of older workers [20,21], with repercussions on their inclination to prolong the working life of older employees. In some instances, this could relate to individual characteristics that cannot be changed, such as age and gender, or are difficult to change in older age, such as the level of qualification [22]. For this reason, van Dalen, Henkens, and Schippers [23] pointed out that research should pay particular attention to employers’ attitudes and behavior concerning different categories of employees. Companies relying to a large extent on high-skilled workers are more likely to retain older employees, according to the human capital hypothesis [16]. The assumption is that highly skilled workers both hold key company knowledge and experience, and they acquire new skills more easily. They both make the extension of their working lives desirable for employers. On the contrary, prolonging the working lives of low-skilled workers who did not retire could be less desirable for employers. However, employers could find it useful to hire retired low-skilled individuals for low-paid part-time occupations [24], often implying a lower social security contribution burden for employers [25]. Moreover, having a large share of older employees that are close to retirement age implies that ways for retirement transitions without labor shortages have to be found. Therefore, the staff´s age profile could be correlated with the company’s will to prolong working lives [26]. As regards employees’ gender, cumulative stratification theories [27] suggest that the unequal allocation of opportunities and resources over the life course increase financial inequality. This may influence decisions regarding retirement, with women being more likely to extend working life than men, since they are less able to build pension income due to their family caregiver role [1].

**H2a:**  *Companies with a high share of older workers, high-skilled or female workers are more likely to extend the working life of older employees.*

**H2b:**  *Companies with a high share of low-skilled workers are more likely to extend the working life through the recruitment of retired former employees.*

#### 1.1.3. Structural Characteristics of the Company

It is sensible to differentiate between sectors, since the workload and age-productivity profiles differ between sectors, and solutions should differ accordingly [5]. For instance, finding skilled workers in the industry sector might be difficult, so manufacturing companies could be more interested in extending workers’ professional lives [28]. Public sector organizations seem to be more conscious of age management issues than companies in other sectors [28]. Yet, given that employment in the public sector is usually strongly regulated, it is likely that unretirement is less widespread there. EWL practices in different sectors are being explored since it is essential to target age management efforts to the different needs, problems, and challenges facing different sectors [29].

Company size also matters. Most studies show that age management initiatives are more widespread in larger companies, which is also due to the fact that HR management is more developed in larger companies than in smaller companies [5,8].

**H3a:**  *Larger companies are more likely to extend the working life of their employees.*

**H3b:**  *Employers in the industry sector and in the public sector are generally more likely to extend working lives than employers in the service sector. Furthermore, unretirement is less widespread in the public sector than in the industry sector.*

#### 1.1.4. Position of the Company in the Larger Economic and Labor Market Climate

A company’s behavior might also be linked to circumstances, such as the company-specific economic situation and the interplay with the economic climate/business cycle and with social partners, for instance, unions. Business cycle theory deals with observable fluctuations in major economic variables (e.g., Gross Domestic Product (GDP), employment rate, etc.) in order to more or less understand regular economic oscillations [30]. This also reflects in alternate periods of good trade with high prices, and periods of bad trade with falling prices. All of this affects choices that are made by employers [31]. In times of economic crises, employers are strongly focused on cost-saving policies [2] and mostly inclined to dismiss older workers [32]. In times of economic growth, employers are inclined to implement HR policies that favor EWL, e.g., due to the good order situation and possible recruitment problems [26]. Trade unions is another important factor. In many countries, their representatives complain that policy reforms force older people to remain in employment longer [33], and highly unionized companies may find it difficult to extend workers’ professional lives [28]. Additionally, job requirements are important aspects to consider. This is especially true of companies operating in a dynamic environment, in which knowledge changes rapidly and there is a need to keep workers up-to-date and innovative. Consequently, when knowledge intensity is high, the company may be more likely to invest in its older employees, thus maintaining the organization-specific knowledge that is embedded in the most experienced staff members [26].

**H4a:**  *Companies needing to reduce staff and/or with high labor union involvement are less likely to extend the working lives of their employees.*

**H4b:**  *Companies facing recruitment problems and/or high knowledge intensity are more likely to extend the working lives of their employees.*

Most of the studies concerning employers’ behavior towards older workers are single-country studies with country-specific results [9,31,34]. However, the wider economic, structural, political, and legal context in each country implies country differences, thus affecting the nature of HR policies. This study relies on a European database involving companies from six countries belonging to different welfare regimes in order to understand the differences between countries [35,36,37,38]: social democratic (Denmark and Sweden), social democratic-liberal (the Netherlands), and conservative (Germany), involving high employment rates of older workers above the EU28 average (in 2018, from 77.9% in Sweden to 67.7% in the Netherlands), besides the Mediterranean or Southern Italy and post-communist (Poland) regimes, being characterized by lower employment rates of older workers, with 53.7% in Italy and 48.9% in Poland [39].

## 2. Materials and Methods

### 2.1. Study Design

Data are drawn from the research European project ‘Activating Senior Potential in Aging Europe’ (ASPA), which was funded within the EU 7th Framework Research Program under the Socio-Economic Sciences and Humanities theme (2008–2011). Data collection took place in 2009. This study includes 4624 companies with at least ten employees in six European countries: Denmark (587), Germany (666), Italy (770), the Netherlands (1046), Poland (1030), and Sweden (525). In each country, the sample was stratified by size and sector. Sector classification is based on the Statistical Classification of Economic Activities in the European Union (Nomenclature statistique des Activités économiques dans la Communauté Européenne (NACE)) [40], and the sectors were categorized according to industry (NACE B–F), (private) services (NACE G–N), and public sector (NACE O–Q). Large companies were overrepresented in the sample to include a sufficient number. Agricultural companies were excluded, since they are mainly composed of self-employed people. The interviews were carried out with an identical questionnaire across countries. The interview techniques differed between countries, depending on country-based methodological considerations [26]: computer-assisted telephone interviewing—CATI (Italy and Poland); computer-assisted web interviewing—CAWI (Denmark); and, paper and pencil interviewing—PAPI (Germany, the Netherlands, and Sweden). The interview duration was about 40 min, on average. The sample of companies in each country was selected from national databases (e.g., the Register of Companies in the Chamber of Commerce). The response rates varied between 11% (Germany) and 53% (Sweden), and they were generally in line with the response rates of surveys carried out among employers [41]. The respondents were specifically selected on the basis of holding high positions in the company and/or managing HR policies and practices within the organization. This information was requested at the beginning of the interview. These respondents (one respondent for each company) were directors/CEOs/owners (29%); heads of departments (10%), general managers (15%), HR managers (31%), and administrators/other positions (15%).

### 2.2. Measures

Dependent variables. The labor demand policies were measured through three different variables in response to a question concerning which of the following measures are currently applied in the organization (Yes/No):

a) Encouraging employees to continue working until the statutory retirement age (i.e., no early retirement).
b) Encouraging employees to continue working beyond the statutory retirement age (i.e., delay of retirement).
c) Recruiting employees who already retired (i.e., unretirement).

While it is clear that the specific intervention referring to the third dependent variable (i.e., unretirement) concerns recruiting retired people, in the case of the other dependent variables the dataset did not allow for having specific examples regarding how employers are encouraging older workers to continue working until or beyond retirement age. Examples of this, among possible others, could be interviews/conversations during which the employer asks and encourages older workers to delay retirement. In this respect, employers can explain to older workers why their work is beneficial to the company, or can provide economic incentives to stay in employment. Employers may also improve working conditions and the working environment (e.g., in terms of tasks, special regulations concerning shift work and holidays, and work burdens). All of this can increase older workers’ satisfaction at work and their willingness and ability to work longer.

Independent variables. With regard to the measurement of HR policies, a list of measures was presented to employers, and these were asked to indicate whether their organization was currently applying them (Y/N). The list was based on earlier research into age-conscious personnel policies e.g., [8,42]. The measures that are investigated in this study are: part-time retirement, early retirement, decreased workload, demotion, and training plans. The following company workforce characteristics were investigated: percentages of high-skilled workers, low-skilled workers, older workers, and female workers. Size and sector were stratification factors. The following questions were asked with regard to the impact of the economic and labor market climate: the extent to which the establishment encounters the need to reduce the staff levels (answer categories: no/low extent; some extent/high extent); whether the company experienced recruitment problems in the last two years (Y/N); the extent to which the respondent agreed or disagreed with the following statements: “Knowledge intensity in our establishment is high”; and, “Labor union influence on personnel policies is clearly visible in the establishment” (five answer categories ranging from “completely disagree” to “completely agree”). The country was included, among other independent variables.

### 2.3. Sample Description

Table 1 presents the main characteristics of the sample. The most widespread labor demand measure is encouraging employees to work until their statutory retirement age (28.9% of the total sample), which is particularly present in Danish and Polish companies. As regards the recruitment of retired people, 17.6% of the companies adopt this strategy, especially companies operating in Poland, where, despite and/or because of the low employment rate of older workers in comparison to most European countries [39], hiring pensioners is a widely used strategy by companies. In Italy, there is a scarce dissemination of measures. Early retirement is still a widespread organizational policy (30% of the companies), particularly in the Netherlands, where part-time retirement and lower workloads for older workers are also frequently adopted. Demotion was more usual in Danish companies, while training plans for older workers were more common in Polish and German ones.

Polish (37.8%) and Swedish (36%) companies had the highest share of high-skilled workers, on average, and Swedish (37.3%) and Italian (36%) companies the highest share of low-skilled workers. On average, 25% of the companies’ workforce comprised older workers, with the highest values in Sweden (31.2%) and the lowest in Italy (19.3%). The workforce gender composition is in favor of men, with the highest share of women, on average, being employed in German companies (49.8%). Overall, 36.3% of the investigated companies were small, 35.7% were medium-sized, and 28% large.

With reference to the sectors, 35.9% of the companies were in the industry sector, 29.8% in services, and 34.3% in the public sector. Approximately 30% of the companies encountered the need to reduce staff levels to a certain or high extent, particularly in Sweden (45%). In particular, Dutch (57.4%), German (56.3%), and Danish (55.5%) companies had recruitment problems in the last two years, but much less so in the case of Italian firms. Knowledge intensity was high, particularly in Polish and Swedish establishments, while the involvement of labor unions was higher in the Swedish and Italian companies.

### 2.4. Analyses

Analyses were carried out for the total sample and for the three sectors separately. Multiple logistic regression models were used to test the variables that were associated with the implementation of measures to extend working life while controlling for other factors. In the total sample model, the sector was included among other regressors. In multivariate analyses within sectors, Average Marginal Effects (AMEs) were employed that consider unobserved heterogeneity across groups in order to allow a comparison of probability values across sectors [43]. A probability value of less than 0.05 was considered for statistical significance. The data were analyzed while using SPSS 23 (SPSS Inc., Chicago, IL, USA) and STATA 11.2 (StataCorp, College Station, TX, USA).

## 3. Results

Table 2 concerns the results of encouraging employees to work until retirement age (i.e., prevention of early retirement) in terms of AMEs for the three sectors. If we take the “industry” model as an example, with the “part-time retirement” variable, the AME represents the change in the probability that companies implementing part-time retirement (Yes) will encourage working until retirement age, when compared to the reference category (No). In this case, companies implementing part-time retirement have a 6% higher probability of encouraging working until retirement age than companies that do not implement part-time retirement. AME represents a good approximation of the change in probability when there is a one-unit change in the independent variable in the case of continuous variables (e.g., in the case of percentage of older workers, one percentage point). Sector-specific models show some differences across sectors in terms of predictors, despite the sector not being significant in the total sample model.

The implementation of HR policies for older workers shows a positive association with encouraging employees to work until legal retirement age. The analyses within sectors show that, for part-time retirement, this positive link only occurs in industry, while training plans do not seem to have a role in the public sector. In the services sector, not surprisingly, the adoption of early retirement schemes was negatively associated with encouraging employees to work until legal retirement age. Having a high share of older workers was positively associated with encouraging employees to work until retirement age, while having a high percentage of high-skilled workers is positively associated with the outcome variable only in industry. Large companies are more likely to implement this measure, but this does not seem to concern industry. Encouraging employees to work until legal retirement age is, plausibly, negatively associated with the need to reduce staff levels, but this only occurs in industry (and in the total sample). This measure is positively associated with recent recruitment problems, although this does not seem to concern services. The presence of knowledge intensity in the firm is associated with encouraging employees to work until legal retirement in the public sector. All countries differ from Sweden, with the exception of Germany. In Denmark (in the industry sector) and Poland (apart from the services sector) encouraging employees to work until retirement age seems to be the case more than in Sweden. The opposite seems to be true in the Netherlands (not in industry) and Italy (apart from industry).

Table 3 shows the results for encouraging employees to work beyond the legal retirement age. The sector was not significant in the total sample model; however, several differences regarding predictors between the sectors were found in the comparison of models within sectors.

HR policies have positive associations with encouraging employees to remain in the company beyond retirement age. An exception to this is related to early retirement schemes, which have a negative link with this measure in the services sector. The positive association concerns part-time retirement (industry and total sample), decreasing the workload (apart from industry), and demotion (apart from services). With regard to training plans, this positive association was found in the total sample and in the services sector. A high share of older workers in a company is also associated with this type of EWL (not in industry). While having a high share of low-skilled workers is positively associated with encouraging employees to work beyond retirement age, this is not the case in the models within sectors. Company size does not seem to play a role. The need to reduce staff levels is negatively associated with the dependent variable apart from in the public sector, while having experienced recruitment problems explains encouraging employees to work beyond the retirement age in industry (and in the total sample).

Companies are more likely to encourage work beyond retirement age in Poland (apart from the public sector) and Denmark (in industry). By contrast, in the Netherlands, Germany, and Italy, companies are less likely to do this than in Sweden. In analyses within sectors, this happens in the public and the services sector in Germany and in Italy, and in the public sector in the Netherlands.

Table 4 reports the results for the recruitment of employees who are already retired. The sector was not significant in the total sample model, but different predictors between sectors were also found in this case.

Part-time retirement (not in the public sector) and workload reduction (not in industry) are among the HR policies that are positively associated with unretirement. Interestingly, the implementation of early retirement schemes was positively associated with recruiting retired people in industry (and in the total sample). Regarding workforce characteristics, a high percentage of older workers was associated with unretirement. On the other hand, high percentages of high and low-skilled workforces played a role in services and industry, respectively. This measure is fostered by company size (larger) and recruitment problems (not in services). For this specific type of labor demand, knowledge intensity also contributed in industry, while, in this sector (and in the total sample), the unions’ strong influence was negatively associated with unretirement.

In most countries, the recruitment of retired people is more widespread than in Sweden. This is prevalent in Poland, and also in Denmark, Germany, and Italy, albeit with some differences across sectors. In the Netherlands and Italy, unretirement is less of an option in the public sector.

## 4. Discussion

The discussion of the main results is structured according to the formulated hypotheses. It will concern the total sample and, when relevant, differences across countries. Finally, the main country differences are discussed.

### 4.1. Role of Human Resource Policies

In line with the human capital theory [16], HR policies for older workers to boost their productivity were positively associated with the three different forms of labor demand, thus supporting H1a, with few exceptions. There are differences across sectors: part-time retirement seems to be linked to EWL particularly in industry, where work is usually physically harder than in other sectors. Nevertheless, industry companies seem to be less likely to promote workload reduction than companies in other sectors. Consistent with past research [44], this might indicate that the hard physical work of older workers in industry could be managed through less working time or a slower pace, rather than through moving these workers to lighter tasks. Training plans for older workers seem to prevail less on EWL practices in the public sector. The reason for this should be further investigated, while considering that older workers are overrepresented in this sector [45], while being generally underrepresented in further training [46]. Basically, older workers´ training biographies and preferences can markedly differ from those of younger workers, so that different forms of (especially non-frontal) training for older workers can be suitable [47]. Policy implications in this respect concern to increase awareness at the company level of the positive aspects (e.g., productivity, job satisfaction, labor supply) of managing older workers through specific HR practices, by specifying that these policies should be differentiated according to the sector of activity of the companies, which imply different needs at the company level. The policy level should encourage age management practices through specific investments.

Hypothesis 1b was also supported: the implementation of early retirement schemes was associated with unretirement. This might suggest that, in some cases, early retirement schemes could be used by companies to re-hire older former workers with valuable skills, thus saving on costs [19]. On the one hand, this practice could be considered to be detrimental to older workers, resulting in their earning a lower income than the one earned prior to retirement (e.g., lower salary and a reduction in working hours). Indeed, early retirees are more likely to have retired involuntarily than older retirees and, for this reason, they may also be more interested in returning to work [48]. On the other hand, older workers, who, by summing up their earnings from their pension and work, which is possible in several European countries, could earn an overall income that is higher than their earnings prior to retirement, could also appreciate this option [25]. This also considering that, in some countries, labor market exit is necessary to receive pension payments [48]. Therefore, there are elements for arguing that, in some cases, this relationship could be beneficial to both employers and employees. The positive link between early retirement and unretirement turned out to be valid, especially in industry. It is likely that industry companies, especially, may try to save costs in this way, while early retirement is atypical in the public sector [25]. Policy action in this regard is expected to protect both older and younger workers. With respect to the former, tools should be employed to prevent them from a possible undesired situation (i.e., dismissal and new recruitment). Instead, the latter could experience increased difficulty to access the labor market due to the unretirement phenomenon. This is the case, especially if older workers have an advantage due to lower social security contributions. For example, in Germany, for workers above legal retirement age, no contributions to the unemployment insurance have to be paid. Older workers have a competitive advantage, since employers partly pay these contributions. Thus, the recruitment of formerly retired employees could be linked, at the policy level, to initiatives for increasing the employability of younger workers, especially in the industry sector.

### 4.2. Workforce Characteristics: Stereotypes and Economic Considerations

Stereotypical views of employers regarding groups of workers were found to influence employers’ inclination towards EWL [20]. Consistent with hypothesis (H2a), we found that having a high share of older workers was positively associated with all three kinds of labor demand. Rather than being specifically linked to stereotypes, in this case, this might mean that these companies try to prevent labor shortages [28]. Yet, hypothesis (H2a) is only partially supported, since there was no evidence of the expected association between a high share of highly skilled workers or of female workers and labor demand practices in the whole sample. However, highly skilled workers were found to be encouraged to work until retirement age by industry sector employers, and to be recruited after their retirement by services sector employers.

Hypothesis (H2b) was supported: a high share of low-skilled workers fosters encouraging work beyond retirement age and unretirement. The latter could be an attempt by employers to reduce costs, also in light of stereotyped negative views regarding productivity [20], through low levels of remuneration or social security contributions [24]. According to the results of this study, this might especially apply to industry sector employers. Based on these results, the policy level should reflect the fact that initiatives at the company and at the governmental level should concentrate on improving working conditions of older workers, in order to add quality to quantity, since the share of older workers in companies is not only increasing, but older workers are also staying in employment longer. While this could be easier in the case of highly skilled workers, it is especially on low-skilled older workers that these efforts could focus.

### 4.3. Company Size and Sector

In line with Hypothesis (H3a), a large company size, in general, increases the likelihood of labor demand practices. However, in general, these companies seem to prefer to stop the work contract once employees reach retirement age and then to re-hire them later, which reduces costs, as discussed above (i.e., 4.1.). The latter seems to apply less in industry, where unretirement, and thus possibly strategies to manage the costs-productivity dilemma, is more likely in medium-sized companies.

Hypothesis (H3b) was not supported, since, once considered the total sample, the sector did not affect the probability of the three kinds of labor demand. This might be due to the fact that the legal frameworks at the macro level regulating work in older age and the transition to retirement do not change across sectors. However, what is interesting, and how we have found in this study by investigating individual sectors, is that the predictors of EWL practices in many cases vary across companies’ activity sectors.

### 4.4. The Economic and Labor Market Climate

Hypothesis 4a was mostly supported. The need to reduce staff was negatively associated with labor demand practices, in line with business cycle theory [30] and Conen and colleagues [31]. Interestingly, our results highlight that this is not an issue concerning unretirement, since it may be easier to hire pensioners to save costs. The mentioned negative association was not evident in the public sector. This is not surprising, since, in times of economic crises, and with legal restrictions to early retirement, the preferred option to reduce costs is usually a hiring freeze, i.e., employees leaving the organization are not replaced [49]. For unretirement, the influence of unions is, as expected, negatively associated with this practice, which is in line with unions´ position on this issue [33]. Industry plausibly confirms the latter, a sector in which unions are strong.

As hypothesized, (H4b) results suggest that employers try to extend older workers’ professional lives in the case of recruitment problems (see also van Dalen and colleagues [26]). Recruitment problems do not lead to EWL practices in the services sector, which suggests a possible preference for recruiting younger workers despite these problems. One explanation for this might be that age stereotypes are particularly present in jobs that require a direct interaction with customers, as is commonly the case in the services sector. Older workers may be moved to back-office activities or are less desirable, since they are thought to resist innovation and ICT tools [50,51]. High knowledge intensity in the company seems to be mainly linked to the recruitment of retired former employees, being plausibly driven by these workers’ relevant knowledge, and especially industry sector employers may be willing to continue to use the knowledge of former workers in this way.

These results could mean that, in the private sector in times of economic difficulties, companies are mostly concentrated on saving costs through EWL practices, while older workers’ needs could take second place. In these times, action at the macro policy level could imply concertation, together with social partners, in order to overcome difficulties by reducing the negative impacts at both the company and workforce levels. Instead, in the event of the company needing to invest (in terms of recruitment or of retaining experience), the services sector seems to be the less available to do so by valorizing older workers. Thus, awareness campaigns in this respect should be especially addressed to employers operating in this sector, by ensuring that, in this sector, older workers may continue to contribute at their best, and be engaged in the working environment.

### 4.5. Country Differences in EWL

Within a common European trend of increasing the employment of older workers, employers behave differently at the national level with regard to extending the working lives of older workers due to pressure from European policy and national pension system reforms extending the working life [34]. The results of this study demonstrate that, in the social democratic regime [35], i.e., in Sweden and Denmark, EWL practices are common. Social democracy is a determinant of employment-friendly and activation policies, for example, flexicurity, through which it is relatively easy to avoid long unemployment. Moreover, the social democratic regime is oriented towards keeping employed individuals in the labor market through social policy investment in specific skills, which, in turn, drives workers and employers’ investment in these skills [52]. According to the results, despite social democratic elements in the Dutch regime [38], Dutch employers seem to be less engaged in extending their employees’ working lives. As in Sweden and Denmark, welfare state reforms support labor market flexibility; however, the main obstacles to EWL identified in this country are very low chances of re-employment and a strong early-exit culture by employers. The latter can be observed in employers’ lack of support towards EWL [53]. In line with Hokema and Lux [54], in the German conservative regime, which is characterized by a strong public social insurance system and a limited tradition in active labor market policies [35,36,37,38,39,40,41,42,43,44,45,46,47,48,49,50,51,52,53,54,55], employers seem to be mostly interested in recruiting employees who already retired. Their limited efforts in encouraging older workers to work until retirement age may be explained by the abolition of early retirement schemes in this country [56], while, after retirement, employers can aim to hire former workers with a lower number of working hours, which is advantageous to employers in terms of social security contributions. This still improves employees’ income situation [57], also in light of the fact that German retirees can earn as much as they want to without their pension being reduced. This is not surprising with reference to the result of Italian employers, who are less willing to prolong the working life of employees than in other countries. In Italy, a country that is characterized almost totally by SMEs, active labor market policies mainly address young people to promote generational turnover [58]. Moreover, this aim is pursued through flexibility in the labor market, which makes it unstable to the extent that the term “flexinsecurity” was coined to describe it [59]. Thus, despite increases in retirement age in the last decades, there is limited support for employers at policy level favoring EWL. Consequently, employers are still oriented towards a culture of early exit programs and they are not inclined to prolong the working life of older workers, partly due to a negative view concerning the cost-productivity balance [25]. This view does not conflict, in the perspective of Italian older workers, with the fact that they would like to retire as soon as possible [60]. A partly surprising result is that Polish employers, in a country that is similar to Italy with a low employment rate of older workers are, by contrast to Italian employers, considerably engaged in prolonging the working life of older workers. However, the issue of population aging has gained importance among policy makers in Poland. The Ministry of Family, Labor, and Social Policy regularly monitors levels of active aging in different domains, including employment [61], since the policy level is strongly committed to reducing unemployment rates and increasing the employment rates of older workers [62]. Accordingly, to some extent, the engagement of Polish employers in EWL practices can be read along these lines, confirming the already observed proactive role of Polish employers in this field [62].

We did not have the possibility to study countries belonging to the liberal regime due to the characteristics of the dataset employed in this study (e.g., the UK or the US). However, since we found differences between the European countries under investigation, it is likely that employers of companies operating in the liberal regime would behave with their own peculiarities with respect to EWL practices. The latter is also in the light of the particular legal framework in this field existing in countries that belong to the liberal regime, which is strongly oriented towards pro-work incentives for older workers. For instance, mandatory retirement was eliminated in 1986 in the US for the majority of workers, while this was done in the UK in 2011 [60], with this meaning that, in these countries, older workers could have more power of decision about EWL than older workers of other countries. Congruently, the UK is one of the European countries with the highest increase in employment beyond retirement age, being this mostly a matter of low-skilled workers [47] However, despite this, for instance, in the UK, there are still concerns regarding the readiness of employers to deal with workforce ageing and with managing EWL [63]; therefore, future research would be needed to better understand employers’ attitudes and behaviors towards EWL in the liberal regime.

It is also important to reflect on the fact that the data employed in this study were collected some years ago, and it is fundamental to understand how the main changes occurred in the last years about the topic under investigation at the policy and economic levels, would relate to the results obtained here. At the policy level, in the last decade, policy efforts to increase retirement age continued at both the European and the level of national governments. Between 2009 and 2018, the employment rate of older workers increased in all the countries under study: from 18.1 percentage points (PPs) in Italy, to eight PPs in Sweden, with the latter country having a very high rate in 2009, already and being still the country, among those under investigation, with the highest employment rate of older workers [39]. In terms of the average effective age of retirement, between the time-spans 2005–2010 and 2013−2018, it also increased for both genders in all of the countries under investigation. The highest increase concerned Italy (+3.3 years for men and +2.5 years for women) and the lowest one Sweden for men (+1 year) and Denmark for women (+0.7 years). Still, the country with the highest average effective age of retirement in the 2018 was Sweden for both men and women (66.4 years and 65.4 years, respectively), while Poland was the country with the lowest one (62.8 years for men and 60.6 years for women) [64]. An important part of these increases might be mostly due to macro-level policies, like national pension system reforms, rather than to companies’ EWL practices (for instance, while we found that Italian employers were less willing to prolong the working life of employees than in other countries, in the last decade Italy evidenced the highest increase, both in terms of employment rate of older workers and of average effective retirement age). Still, the results of this study and the related policy recommendations are useful, since they concern the organizational level and they have to do not only with extending working life in companies, but also with improving employers’ attitudes and behavior towards older workers, making it possible to improve the quality of work, job satisfaction, and productivity. Companies should not feel compelled to rely on older workers due to the existing laws, but rather take advantage of the positive aspects of managing older workers through age management practices, in an active ageing perspective, in order to maintain high productivity standards [65]. This is especially true nowadays, given that the serious negative effects of the 2008 economic crisis have been overcome in most countries and, as the results of this study demonstrate, employers may be more available to invest on older workers in times of healthy economy.

## 5. Conclusions

This paper provides information on the drivers and barriers to EWL in companies, by distinguishing within sector of activity, which was an under investigated research area. This was done by using data from a large European study involving companies from six countries. The results of this study suggest that, among the main predictors of companies’ labor demand for older workers, there is the implementation of HR measures for older workers to improve their performance and working conditions. Other predictors are a high share of older workers, being a medium or large-sized company, and having experienced recruitment problems. There are indications that the possibility of reducing costs through EWL practices is a key element for companies. In the industry sector, the qualities and skills of older workers could be more valued and desired than in other sectors, while, in the public sector, there might be less motivation to retain older workers’ qualities, and the external economic climate could have a milder effect on labor demand aspects concerning older workers. Employers´ engagement in extending the working lives of older workers seems to be high in Sweden, Denmark, and Poland, yet less so in Germany and low in the Netherlands and Italy.

These results have clear policy implications: it is important to push and enable companies to promote more organizational practices to improve the employability and working conditions of older workers through innovative tools. One example of this could be the enforcement of laws, thought out in agreement with social partners, contemplating the possibility to fund and implement, among other active aging measures, these kinds of initiatives in companies [66]. These tools may be used to raise employers’ awareness and increase their actions in companies and sectors that are traditionally reluctant to extend employees’ working lives and/or adopt age management initiatives. Since age management practices are widespread, especially in large firms [34], efforts should concentrate more on SMEs having a low professionalization of HR but a high total number of workers. Policy implications that are based on activity sector relate to, on the one hand, the need to provide opportunities for EWL also in sectors where older workers seem to be less valued, e.g., in the services and public sectors. On the other hand, they concern the need to ensure that, in any case, when EWL practices are provided, the cost-productivity assessment by employers is not to the detriment of older workers. This means that this assessment should not neglect the positive connotation of age management initiatives as opportunities for workers, rather than restrictions [67].

This study’s cross-sectional design is one main limitation. A longitudinal study would have more clearly identified the effect of the independent variables on labor demand, in terms of causality. A second limitation is that the level of country comparison is quite general, while future research should compare countries based on multivariate models run within countries, to obtain a more detailed understanding of country differences. Furthermore, we could not include in the analyses at least one country representing the liberal welfare regime, given the characteristics of the dataset employed. Additionally, the study design made it possible, to a limited extent, to deal with the gender perspective, although there are gender-based inequalities in EWL and in the transition to retirement. We controlled for this through the percentage of the female workforce present in the company (never significant). Another limitation is that data employed in this study have been collected for some years now; however, the results of the study are still relevant and provide useful knowledge on the management of EWL in European companies, as explained in the discussion.

## Figures and Tables

**Table 1:** Sample characteristics, by country and total sample, % mean (SD).

	Sweden	Denmark	Netherlands	Germany	Italy	Poland	Total
Numbers of Cases	525	587	1046	666	770	1030	4624
**EWL in companies (yes)**							
Encour\aging working until statutory retirement age	28.6	42.8	20.9	27.7	10.8	44.1	28.9
Encouraging working beyond retirement age	14.3	21.2	8.5	6.8	6.1	29.0	15.0
Recruiting employees who already retired	7.8	12.8	8.6	14.6	3.9	43.1	17.6
**HR Policies (yes)**							
Part-time retirement	24.6	19.7	49.9	16.4	4.5	28.3	25.7
Early retirement schemes	19.0	15.1	55.0	23.3	10.1	39.1	30.0
Decreasing the workload for older workers	13.5	36.0	37.0	8.4	7.4	9.9	18.8
Demotion	3.4	21.2	8.4	4.3	0.8	2.9	6.3
Training plans for older workers	11.0	11.5	14.9	24.9	2.2	39.5	19.1
**Company’s workforce characteristics**							
% high-skilled workers	36.0 (33.9)	20.0 (24.8)	18.6 (27.0)	19.0 (23.5)	26.0 (29.8)	37.8 (31.8)	26.0 (29.6)
% low-skilled workers	37.3 (34.5)	23.0 (26.9)	18.2 (26.8)	15.9 (20.0)	36.0 (35.5)	17.4 (23.4)	23.1 (28.8)
% older workers	31.2 (18.9)	26.7 (15.1)	23.3 (15.6)	26.8 (15.2)	19.3 (18.5)	25.3 (19.7)	25.0 (17.6)
% female workers	42.6 (28.7)	41.2 (25.7)	35.0 (27.3)	49.8 (27.3)	40.3 (27.6)	45.7 (28.8)	42.3 (28.0)
**Company’s structural characteristics**							
Size							
*Small*	38.3	32.7	33.8	28.4	37.9	44.7	36.3
*Medium*	32.6	33.0	34.2	34.3	34.5	41.4	35.7
*Large*	29.1	34.3	32.0	34.6	27.5	13.8	28.0
Sector							
*Industry*	36.7	35.3	35.7	25.0	40.4	41.7	35.9
*Services*	28.8	30.9	33.1	24.4	31.7	28.9	29.8
*Public*	34.5	33.8	31.2	48.9	27.9	29.3	34.3
**Economic and labor market climate**							
Need to reduce staff to some/high extent	45.0	33.0	36.0	26.3	32.1	14.9	29.9
Recruitment problems (yes)	26.7	55.5	57.4	56.3	28.8	35.9	44.9
Knowledge intensity (1—low, 5—high)	4.0 (0.82)	3.8 (0.89)	3.7 (0.96)	3.9 (0.76)	2.1 (0.84)	4.1 (0.80)	3.6 (1.07)
Labor unions involvement (1—low, 5—high)	3.3 (1.11)	2.8 (1.23)	2.6 (1.18)	2.3 (1.21)	3.1 (1.26)	2.5 (1.40)	2.7 (1.28)

**Table 2:** Explanatory variables for encouraging working until statutory retirement age, by sector and total sample.

	Industry			Services			Public			Total Sample	
Number of Cases	1379			1126			1265			3938	
	AME	SE	*p*-Value	AME	SE	*p*-Value	AME	SE	*p*-Value	B	*p*-Value
**HR Policies** (yes)											
Part-time retirement	0.06	0.03	**0.042**	0.04	0.04	0.283	0.04	0.03	0.198	0.29	**0.004**
Early retirement schemes	0.01	0.03	0.756	−0.06	0.03	**0.041**	−0.01	0.03	0.793	−0.09	0.373
Decreasing the workload for older workers	0.12	0.03	**0.000**	0.12	0.04	**0.002**	0.11	0.03	**0.001**	0.59	**0.000**
Demotion	0.16	0.05	**0.005**	0.09	0.05	0.056	0.17	0.06	**0.002**	0.73	**0.000**
Training plans for older workers	0.08	0.03	**0.026**	0.11	0.04	**0.004**	0.05	0.03	0.083	0.41	**0.000**
**Company’s workforce characteristics**											
% high-skilled workers	0.00	0.00	**0.013**	0.00	0.00	0.981	0.00	0.00	0.767	0.00	0.102
% low-skilled workers	0.00	0.00	0.414	0.00	0.00	0.686	0.00	0.00	0.085	0.00	0.117
% older workers	0.00	0.00	**0.037**	0.00	0.00	**0.000**	0.00	0.00	**0.004**	0.01	**0.000**
% female workers	0.00	0.00	0.357	0.00	0.00	00.153	0.00	0.00	0.627	−0.00	0.500
**Company’s structural characteristics**											
Size (ref. Small)											
*Medium*	0.00	0.03	0.947	−0.02	0.03	0.479	0.05	0.03	0.082	0.07	0.463
*Large*	−0.01	0.03	0.816	0.09	0.04	**0.011**	0.06	0.03	0.060	0.28	**0.010**
Sector (ref. Industry)											
*Services*	-	-	-	-	-	-	-	-	-	0.01	0.907
*Public*	-	-	-	-	-	-	-	-	-	−0.09	0.491
**Economic and labor market climate**											
Need to reduce staff to some/high extent	−0.06	0.02	**0.014**	−0.05	0.03	0.083	−0.01	0.03	0.730	−0.21	**0.019**
Recruitment problems (yes)	0.08	0.02	**0.002**	0.04	0.03	0.115	0.09	0.03	**0.001**	0.41	**0.000**
Knowledge intensity	0.02	0.01	0.214	−0.01	0.01	0.443	0.04	0.02	**0.047**	0.08	0.093
Labor unions involvement	−0.01	0.01	0.563	−0.01	0.01	0.204	−0.01	0.01	0.339	−0.06	0.077
**Country** (ref. Sweden)											
Denmark	0.16	0.05	**0.002**	0.00	0.06	0.955	0.02	0.06	0.728	0.39	**0.011**
Netherlands	−0.08	0.04	**0.079**	−0.10	0.05	**0.044**	−0.19	0.05	**0.000**	−0.67	**0.000**
Germany	−0.05	0.05	0.292	−0.03	0.06	0.609	−0.09	0.05	0.099	−0.19	0.235
Italy	−0.05	0.05	0.335	−0.20	0.05	**0.000**	−0.20	0.06	**0.001**	−0.79	**0.000**
Poland	0.13	0.05	**0.005**	0.07	0.06	0.249	0.13	0.06	**0.024**	0.60	**0.000**
R-squared	0.12			0.13			0.12			0.18	

Multiple logistic regression on encouraging working until statutory retirement age (Y/N). Average Marginal Effects (AMEs) represent the probability of discrete change from the base level. Statistically significant associations in bold. SE—standard error; B—beta value.

**Table 3:** Explanatory variables for the encouraging working beyond retirement age, by sector and total sample.

	Industry			Services			Public			Total Sample	
Number of Cases	1379			1121			1267			3933	
	AME	SE	*p*-Value	AME	SE	*p*-Value	AME	SE	*p*-Value	B	*p*-Value
**HR Policies** (yes)											
Part-time retirement	0.06	0.03	**0.036**	0.04	0.03	0.198	0.03	0.02	0.177	0.33	**0.007**
Early retirement schemes	0.00	0.02	0.856	−0.05	0.02	**0.050**	0.02	0.02	0.294	−0.03	0.789
Decreasing the workload for older workers	0.03	0.02	0.249	0.10	0.03	**0.003**	0.10	0.03	**0.001**	0.57	**0.000**
Demotion	0.11	0.05	**0.014**	0.03	0.04	0.378	0.10	0.04	**0.018**	0.67	**0.000**
Training plans for older workers	0.04	0.03	0.251	0.06	0.03	**0.041**	0.02	0.02	0.443	0.27	**0.029**
**Company’s workforce characteristics**											
% high-skilled workers	0.00	0.00	0.552	0.00	0.00	0.676	0.00	0.00	0.908	0.00	0.362
% low-skilled workers	0.00	0.00	0.322	0.00	0.00	0.087	0.00	0.00	0.648	0.01	**0.017**
% older workers	0.00	0.00	0.259	0.00	0.00	**0.000**	0.00	0.00	**0.018**	0.01	**0.000**
% female workers	0.00	0.00	0.813	0.00	0.00	0.300	0.00	0.00	0.315	0.00	0.897
**Company’s structural characteristics**											
Size (ref. Small)											
*Medium*	0.03	0.02	0.155	0.02	0.02	0.290	0.00	0.02	0.855	0.16	0.207
*Large*	0.00	0.03	0.910	0.02	0.03	0.367	0.01	0.03	0.725	0.14	0.334
Sector (ref. Industry)											
*Services*	-	-	-	-	-	-	-	-	-	0.13	0.301
*Public*	-	-	-	-	-	-	-	-	-	−0.13	0.394
Economic and labor market climate											
Need to reduce staff to some/high extent	−0.05	0.02	**0.005**	−0.04	0.02	**0.041**	0.00	0.02	0.934	−0.30	**0.011**
Recruitment problems (yes)	0.05	0.02	**0.005**	0.02	0.02	0.355	0.02	0.02	0.280	0.32	**0.002**
Knowledge intensity	0.01	0.01	0.371	0.00	0.01	0.946	0.01	0.01	0.457	0.09	0.175
Labor unions involvement	0.00	0.01	0.822	0.00	0.01	0.556	0.00	0.01	0.625	−0.02	0.556
**Country** (ref. Sweden)											
Denmark	0.13	0.04	**0.001**	−0.01	0.04	0.893	−0.01	0.05	0.816	0.31	0.103
Netherlands	−0.02	0.03	0.409	−0.05	0.04	0.174	−0.14	0.04	**0.001**	−0.77	**0.000**
Germany	0.00	0.03	0.922	−0.09	0.04	**0.036**	−0.09	0.04	**0.034**	−0.56	**0.013**
Italy	0.04	0.04	0.286	−0.08	0.04	**0.043**	−0.15	0.05	**0.001**	−0.53	**0.029**
Poland	0.18	0.04	**0.000**	0.11	0.05	**0.032**	0.10	0.06	0.070	0.86	**0.000**
R-squared	0.12			0.14			0.13			0.15	

Multiple logistic regression on encouraging working beyond retirement age (Y/N). Average Marginal Effects (AMEs) represent the probability of discrete change from the base level. Statistically significant associations in bold. SE=standard error; B=beta value.

**Table 4:** Explanatory variables for recruiting employees who already retired, by sector and total sample.

	Industry			Services			Public			Total Sample	
Number of Cases	1358			1128			1271			3952	
	AME	SE	*p*-Value	AME	SE	*p*-Value	AME	SE	*p*-Value	B	*p*-Value
**HR Policies** (yes)											
Part-time retirement	0.05	0.02	**0.044**	0.06	0.03	**0.022**	0.00	0.02	0.961	0.39	**0.002**
Early retirement schemes	0.07	0.02	**0.007**	0.03	0.02	0.186	0.01	0.02	0.596	0.35	**0.003**
Decreasing the workload for older workers	0.03	0.02	0.237	0.07	0.03	**0.022**	0.09	0.03	**0.004**	0.48	**0.000**
Demotion	0.06	0.04	0.142	−0.03	0.03	0.300	0.04	0.04	0.357	0.12	0.510
Training plans for older workers	0.03	0.02	0.274	0.05	0.03	0.071	−0.01	0.02	0.701	0.16	0.170
**Company’s workforce characteristics**											
% high-skilled workers	0.00	0.00	0.406	0.00	0.00	**0.038**	0.00	0.00	0.260	0.00	0.683
% low-skilled workers	0.00	0.00	**0.015**	0.00	0.00	0.799	0.00	0.00	0.363	0.01	**0.032**
% older workers	0.00	0.00	**0.016**	0.00	0.00	**0.000**	0.00	0.00	**0.001**	0.02	**0.000**
% female workers	0.00	0.00	0.735	0.00	0.00	0.134	0.00	0.00	0.907	0.00	0.865
**Company’s structural characteristics**											
Size (ref. Small)											
*Medium*	0.09	0.02	**0.000**	0.08	0.02	**0.000**	0.04	0.02	0.076	0.72	**0.000**
*Large*	0.04	0.03	0.106	0.05	0.02	**0.042**	0.06	0.03	**0.018**	0.55	**0.000**
Sector (ref. Industry)											
*Services*	-	-	-	-	-	-	-	-	-	0.07	0.582
*Public*	-	-	-	-	-	-	-	-	-	−0.18	0.264
**Economic and labor market climate**											
Need to reduce staff to some/high extent	0.02	0.02	0.420	−0.04	0.02	0.056	−0.04	0.02	0.079	−0.11	0.343
Recruitment problems (yes)	0.05	0.02	**0.005**	0.04	0.02	0.066	0.06	0.02	**0.004**	0.46	**0.000**
Knowledge intensity	0.02	0.01	**0.033**	0.02	0.01	0.146	0.01	0.01	0.428	0.18	**0.006**
Labor unions involvement	−0.02	0.01	**0.002**	−0.01	0.01	0.119	−0.01	0.01	0.112	−0.15	**0.000**
**Country** (ref. Sweden)											
Denmark	0.15	0.03	**0.000**	0.01	0.04	0.735	−0.05	0.04	0.240	0.54	**0.020**
Netherlands	0.06	0.02	**0.003**	−0.04	0.03	0.242	−0.09	0.04	**0.010**	−0.30	0.209
Germany	0.18	0.04	**0.000**	0.09	0.04	**0.037**	0.06	0.04	0.174	1.06	**0.000**
Italy	0.06	0.03	**0.040**	0.01	0.04	0.794	−0.10	0.04	**0.015**	−0.05	0.854
Poland	0.34	0.04	**0.000**	0.30	0.05	**0.000**	0.34	0.05	**0.000**	2.18	**0.000**
R-squared	0.22			0.25			0.18			0.26	

Multiple logistic regression on recruiting employees who already retired (Y/N). Average Marginal Effects (AMEs) represent the probability of discrete change from the base level. Statistically significant associations in bold. SE—standard error; B—beta value.
